# Genome-wide methylome analysis using MethylCap-seq uncovers 4 hypermethylated markers with high sensitivity for both adeno- and squamous-cell cervical carcinoma

**DOI:** 10.18632/oncotarget.12598

**Published:** 2016-10-12

**Authors:** Rong Wang, Robert W. van Leeuwen, Aniek Boers, Harry G. Klip, Tim de Meyer, Renske D. M. Steenbergen, Wim van Criekinge, Ate G. J. van der Zee, Ed Schuuring, G. Bea A. Wisman

**Affiliations:** ^1^ Department of Gynecologic Oncology, University of Groningen, University Medical Centre Groningen, Cancer Research Centre Groningen, Groningen, The Netherlands; ^2^ Department of Pathology, University of Groningen, University Medical Centre Groningen, Cancer Research Centre Groningen, Groningen, The Netherlands; ^3^ Department of Laboratory Medicine, Tianjin Medical University, Tianjin, China; ^4^ Department of Mathematical Modeling, Statistics and Bio-informatics, University of Ghent, Ghent, Belgium; ^5^ Department of Pathology, VU University Medical Centre, Amsterdam, The Netherlands

**Keywords:** uterine cervical neoplasms, DNA methylation biomarkers, MethylCap-seq, adenocarcinoma (in situ), (quantitative) methylation-specific PCR ((Q)MSP)

## Abstract

**Background:**

Cytology-based screening methods for cervical adenocarcinoma (ADC) and to a lesser extent squamous-cell carcinoma (SCC) suffer from low sensitivity. DNA hypermethylation analysis in cervical scrapings may improve detection of SCC, but few methylation markers have been described for ADC. We aimed to identify novel methylation markers for the early detection of both ADC and SCC.

**Results:**

Genome-wide methylation profiling for 20 normal cervices, 6 ADC and 6 SCC using MethylCap-seq yielded 53 candidate regions hypermethylated in both ADC and SCC. Verification and independent validation of the 15 most significant regions revealed 5 markers with differential methylation between 17 normals and 13 cancers. Quantitative methylation-specific PCR on cervical cancer scrapings resulted in detection rates ranging between 80% and 92% while between 94% and 99% of control scrapings tested negative. Four markers (*SLC6A5*, *SOX1*, *SOX14* and *TBX20*) detected ADC and SCC with similar sensitivity. In scrapings from women referred with an abnormal smear (n=229), CIN3+ sensitivity was between 36% and 71%, while between 71% and 93% of adenocarcinoma in situ (AdCIS) were detected; and CIN0/1 specificity was between 88% and 98%. Compared to hrHPV, the combination *SOX1*/*SOX14* showed a similar CIN3+ sensitivity (80% vs. 75%, respectively, P>0.2), while specificity improved (42% vs. 84%, respectively, P < 10^-5^).

**Conclusion:**

*SOX1* and *SOX14* are methylation biomarkers applicable for screening of all cervical cancer types.

## INTRODUCTION

Cervical cancer is one of the most common female cancers in the world, with more than 525,000 new cases and over 265,000 deaths occurring globally each year [1, 2]. Cervical squamous-cell carcinoma (SCC) and cervical adenocarcinoma (ADC) are two main histological subtypes of invasive cervical cancer, which account for 75 - 90% and 10 - 25% of cases, respectively [3–5]. Currently, the incidence of SCC is declining in most developed countries. In contrast, there is a rise in the absolute and relative incidence of ADC [6]. In Europe, ADC is increasing rapidly, especially in younger women [7, 8]. In the Netherlands, the absolute incidence rate of ADC increased by 15.8% in women aged 15 - 29 years and 2.5% in women aged 30 - 44 years [7]. Moreover, compared to SCC, ADC is mainly diagnosed in more advanced stages, appears to be less sensitive to (chemo)radiation therapy and is associated with a worse prognosis [9–12].

Both the upward trend in relative incidence as well as delayed detection of ADC are probably due to the present population-based screening programs, which are more effective in the detection of the precursors of SCC than those of ADC. This may be because ADC arises in a more cranial localization in the cervix where it is more difficult to either obtain representative cytology samples or to detect ADC or its precursors by colposcopy [13]. High-risk human papillomavirus (hrHPV) is widely accepted as the predominant etiological agent of cervical cancer [14] and its detection is clinically more sensitive for the detection of cervical adenocarcinoma in situ (AdCIS) and ADC compared to cytology [15]. As hrHPV testing has a relatively low positive predictive value in population-based screening programs [16, 17], women who are tested positive will require an additional test that is equally sensitive to detect SCC and ADC and can also sufficiently detect their precursors to ensure correct referral to the gynecologist for colposcopy [18]. Therefore, novel biomarkers for cervical cancer are required that ideally will identify both ADC and SCC as well as their precursors with high sensitivity.

Aberrant gene expression caused by epigenetic mechanisms is a prominent feature of many types of cancer [19], and DNA promoter methylation of tumor suppressor genes (TSG) has been reported to be an early event in carcinogenesis [20]. DNA methylation markers might be exploited in cancer diagnosis as variations in DNA methylation are observed more frequently than other genetic variations [21]. Although we [22, 23] and others [24] have reported many methylation markers associated with cervical cancer, many of these markers are more frequently methylated in SCC compared to ADC [23]. Moreover, so far only a limited number of methylated genes have been identified that are specifically associated with ADC. Most of these markers have lower sensitivity for both ADC and SCC or either one [25–27].

In the past ten years, advances in whole-genome methylation profiling technologies have revolutionized the field of cancer research. In order to identify cervical cancer-specific methylation markers, approaches such as a pharmacological unmasking expression microarray [28] or immunoprecipitation combined with oligonucleotide microarrays have been performed [29–31]. Nevertheless, microarray-based screening has drawbacks regarding their design and production, and also the inaccurate hybridization signals and variable immunoprecipitation step leave room for further improvement. Reductions in costs have spurred the adoption of next-generation sequencing (NGS) platforms with higher sensitivity and accuracy compared to traditional microarray profiling [32]. Recently, an affinity-based methylation capture assay using methyl-binding domain (MBD) complexes coupled with NGS (MethylCap-seq) has been reported to be an effective technique to comprehensively analyze the methylome in lung cancer, ovarian cancer, head and neck cancer, and high-grade cervical intraepithelial neoplasia (CIN) [33–37]. These technologies have facilitated the discovery of potential DNA methylation biomarkers for disease development and progression as well as our understanding of the complex, underlying molecular mechanisms that lead to cancer.

Until now, no cervical cancer DNA methylome analysis has been performed using ADC. In this study, MethylCap-seq was applied to perform a genome-wide DNA methylation screening of cervical cancer, including both ADC and SCC, and normal cervix tissues. With this approach, we sought to identify genome-wide aberrant methylation patterns of cervical cancer-specific markers with high sensitivity to detect both ADC and SCC and their precursors in cervical scrapings.

## RESULTS

### Identification of methylated candidates by MethylCap-seq

**Figure 1:**
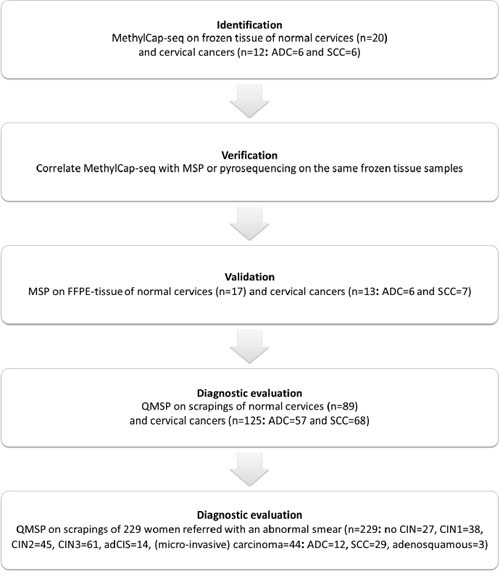
Flow scheme for the identification of new cervical cancer markers

**Figure 2:**
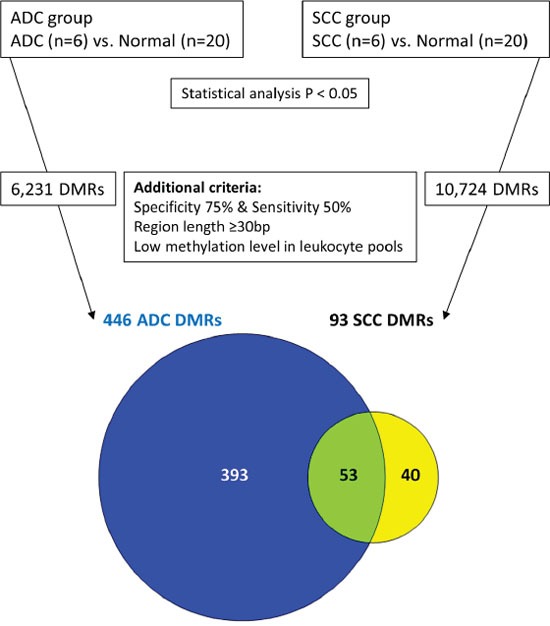
Identification of methylated candidates by MethylCap-seq

**Figure 3:**
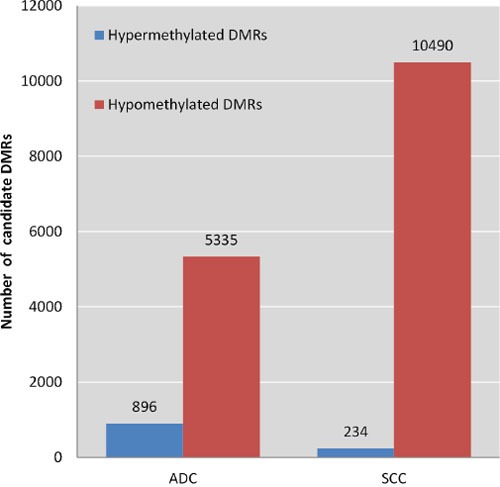
Frequencies of hyper- and hypomethylated regions in ADC and SCC

**Figure 4:**
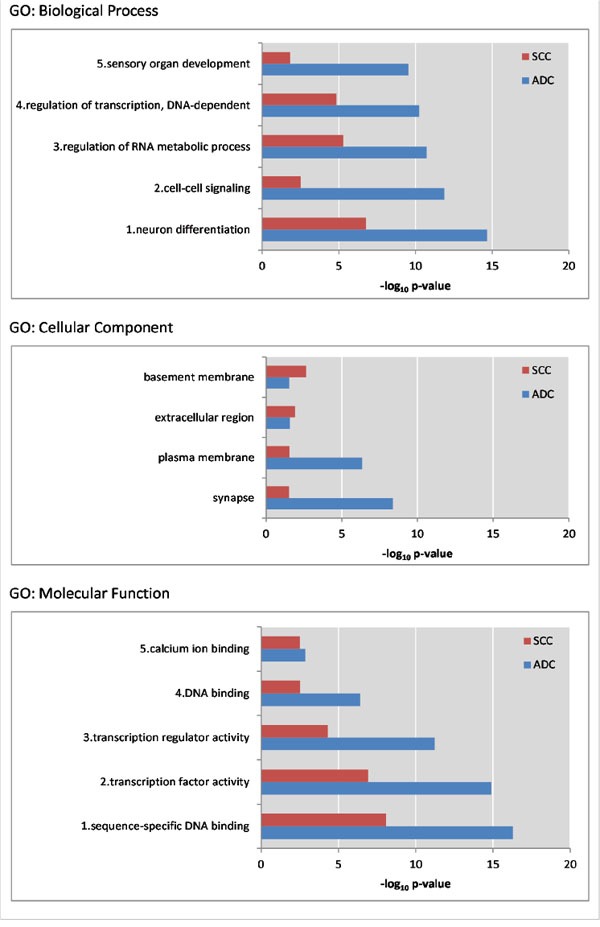
For three GO themes the five most significant GO terms enriched in ADC are shown along with the P-value of that term in SCC Within the cellular component GO terms, only 4 terms were significant in both ADC and SCC. All depicted P-values are below 0.05.

To discover markers that are methylated in both SCC and ADC, but not in normal cervix, we generated a methylome of 6 SCC and 6 ADC and used a stepwise selection approach as outlined in Figure 1. In total, 6,231 candidate differentially methylated regions (DMRs) for ADC compared to normal cervices and 10,724 candidate DMRs for SCC compared to normal cervices were identified after applying our selection criteria (see methods section for detailed description of the identification step, Figure 2). In ADC as well as in SCC hypomethylation was more frequently observed compared to hypermethylation (Figure 3). We focused on the hypermethylated DMRs, as these are more easily translated into methylation-specific PCR (MSP) assays, which can be implemented as clinical diagnostic tests. Overall 446 candidate hypermethylated DMRs, comprising 357 genes, were identified in ADC and 93 DMRs, comprising 89 genes, in SCC. Gene ontology (GO) functional analysis for these DMRs was performed to determine if similar pathways were affected in both cancer histological types. There were in total 328 and 49 GO terms enriched in ADC and SCC, respectively (Supplementary Table S1 and S2). Most GO terms enriched in SCC were also enriched in ADC, as 37/38 of the biological processes, 4/5 of the cellular components and 5/6 of the molecular functions, were also shown in ADC. This observation suggests that similar pathways are disrupted in the carcinogenesis of both histological types. Figure 4 shows the most significant GO terms enriched in ADC together with the associated significance in SCC. We identified 53 regions, comprising 50 genes that were hypermethylated in both ADC and SCC (Figure 2).

### Verification and validation of the top 15 candidates

After identification of 53 candidate regions, the top 15 regions (Supplementary Table S3) were selected for verification of the MethylCap-seq data by either MSP or pyrosequencing analysis. To obtain the top 15 genes, candidate regions were ranked on the number of (i) methylated ADC, (ii) methylated SCC, and on (iii) unmethylated normal cervices. Using the same DNA as used for MethylCap-seq, 10 genes (*SOX1*, *GFRA1*, *SLC6A5*, *TBX5*, *OLIG2*, *AC004963.1*, *TBX20*, *RP11-100E13.1*, *RP1-241P17.1*, and *SOX14*) showed a significant correlation between MSP or pyrosequencing and the number of reads from the MethylCap-seq (Supplementary Table S4).

MSP primers were designed for these 10 markers. Four MSP assays showed high methylation levels in DNA from leukocytes and whole-genome amplified (WGA) DNA and were therefore excluded from further validation (Supplementary Table S4). MSP of the remaining 6 genes was performed on DNA from an independent series of 17 normal cervix and 13 cervical cancer formalin-fixed paraffin-embedded (FFPE) tissue samples (6 ADC and 7 SCC). Except for *TBX5*, all 5 genes (*GFRA1*, *SLC6A5*, *SOX1*, *SOX14* and *TBX20*) were significantly differentially methylated between normal and cancer tissues with a high methylation frequency in both ADC and SCC (Table 1).

**Table 1:** MSP positivity in the external validation cohort of FFPE tissue samples

Gene	Normal		Cancer		ADC		SCC	
*TBX5*	56%	(9/16)	67%	(8/12)	60%	(3/5)	71%	(5/7)
SOX14*	25%	(4/16)	85%	(11/13)	83%	(5/6)	86%	(6/7)
SOX1*	0%	(0/15)	92%	(11/12)	100%	(5/5)	86%	(6/7)
TBX20*	6%	(1/17)	83%	(10/12)	100%	(5/5)	71%	(5/7)
SLC6A5*	7%	(1/15)	83%	(10/12)	100%	(5/5)	71%	(5/7)
GFRA1*	0%	(0/11)	83%	(10/12)	83%	(5/6)	83%	(5/6)

* the positive rates in normal and cancer samples differ (P < 0.05).

### Diagnostic evaluation on scrapings from healthy cervices and cervical cancer scrapings

**Figure 5:**
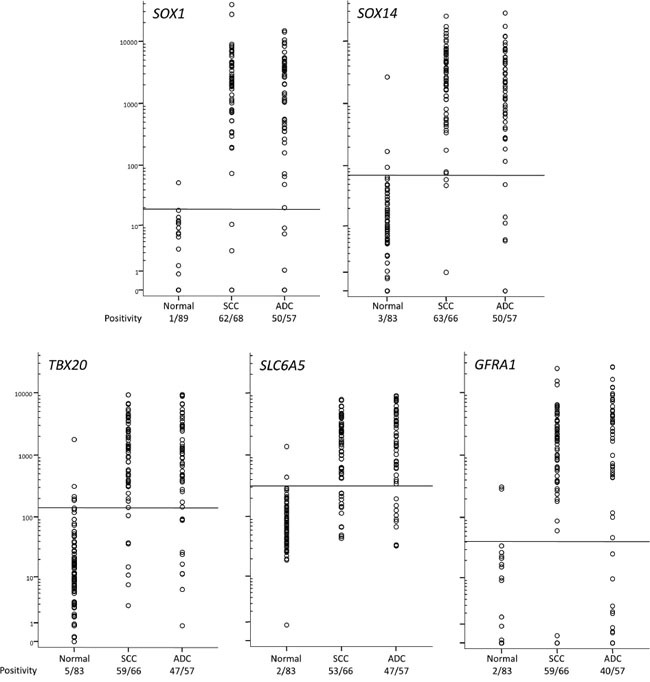
DNA methylation levels in normal and cancer scrapings determined by QMSP The horizontal lines represent optimal thresholds (see Table 2). The positive rate is depicted below the class labels.

QMSP was designed for 5 genes (*GFRA1*, *SLC6A5*, *SOX1*, *SOX14*, *TBX20*) and their diagnostic potential was evaluated on scrapings from a large series of cervical cancer patients (n=125: 57 ADC and 68 SCC) and controls with similar age. The level of DNA methylation for all five genes was significantly different in cancer scrapings compared to normal scrapings (each P < 10^-24^), while methylation levels in ADC and SCC (Figure 5) were similar (all P > 0.10). Because many markers were also methylated in normal scrapings, albeit at lower levels as observed in cancer scrapings, a threshold was set by maximizing Youden's index J using receiver-operator characteristic (ROC) analysis of the individual genes. Hereafter, the specificity and the sensitivity for ADC and SCC were determined for all individual genes (Table 2). The sensitivity of the 5 QMSP assays ranged from 79% to 88%, while the specificity ranged from 94% to 99%. Except for *GFRA1*, all markers (*SLC6A5*, *SOX1*, *SOX14* and *TBX20*) detected ADC and SCC with a similar sensitivity.

**Table 2:** Diagnostic performance of five QMSPs on normal and cancer scrapings

Gene	AUC (95% CI)	J	Cutoff	Positive normal	Positive cancer	P†	Positive ADC	Positive SCC	P‡
*SOX1*	0.96 (0.93–0.99)	0.885	19	1%	90%	10^-37^	88%	91%	0.528
*SOX14*	0.96 (0.93–0.99)	0.883	70	4%	92%	10^-36^	88%	95%	0.118
*TBX20*	0.94 (0.90–0.97)	0.802	140	6%	86%	10^-29^	82%	89%	0.266
*SLC6A5*	0.93 (0.89–0.96)	0.789	315	2%	81%	10^-28^	82%	80%	0.760
*GFRA1*	0.92 (0.88–0.96)	0.781	41	2%	80%	10^-28^	70%	89%	0.007

CI is the confidence interval

† frequency normal vs. cancer

‡ frequency ADC vs. SCC.

### Diagnostic evaluation on scrapings of women referred with an abnormal smear

**Figure 6:**
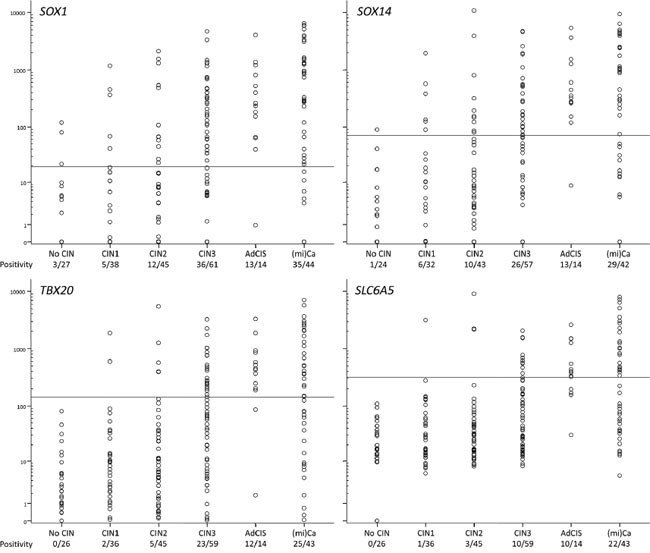
Methylation levels in cervical scrapings of women referred with an abnormal smear The positive rate is depicted below the histological class labels.

In the second diagnostic evaluation 229 scrapings were analyzed for *SLC6A5*, *SOX1*, *SOX14* and *TBX20* hypermethylation using the QMSP threshold values that were set in the normal and cancer samples (Table 2). For each gene both the QMSP ratios and the positive rates increased with the severity of the underlying lesion (P < 10^-9^ for each gene, Figure 6). Also in this cohort no differences in QMSP levels and positivity between ADC and SCC were found (data not shown). Of note, 10 to 13 out of 14 AdCIS scrapings were methylation positive (Figure 6).

Analysis of the sensitivity and specificity of individual genes indicated that *SOX1* was the most sensitive, whereas *SLC6A5* and *TBX20* were the most specific (Table 3, Supplementary Table S5). Next, all possible gene combinations were generated in order to improve the diagnostic performance. The combination with the highest sensitivity and specificity was *SOX1/SOX14*/*TBX20* methylation, in which *SLC6A5* was not additive. The best 2 gene combination was *SOX1/SOX14*.

**Table 3:** Diagnostic performance of individual genes, gene combinations and hrHPV detection in cervical scrapings of women referred with an abnormal smear (ranked on sensitivity CIN3+)

	specificity CIN0/1	sensitivity			P˘
		CIN2+	CIN3+	(mi)Ca	
**Individual genes**					
*SOX1*	88%	59%	71%	80%	5×10^-15^
*SOX14*	88%	50%	60%	69%	8×10^-11^
*TBX20*	97%	40%	52%	58%	2×10^-12^
*SLC6A5*	98%	28%	36%	51%	4×10^-10^
**Gene combinations**					
*SOX1/SOX14/TBX20*	84%	63%	76%	83%	8×10^-15^
*SLC6A5/SOX1/SOX14/TBX20*	84%	63%	76%	83%	8×10^-15^
*SOX1/SOX14*	84%	63%	75%	83%	1×10^-14^
*SLC6A5/SOX1/SOX14*	84%	63%	75%	83%	1×10^-14^
*SOX1/TBX20*	87%	60%	72%	81%	4×10^-15^
*SLC6A5/SOX1/TBX20*	87%	60%	72%	81%	4×10^-15^
*SLC6A5/SOX1*	87%	58%	71%	81%	1×10^-14^
*SOX14/TBX20*	88%	53%	64%	69%	2×10^-11^
*SLC6A5/SOX14/TBX20*	88%	53%	64%	69%	2×10^-11^
*SLC6A5/SOX14*	88%	50%	60%	69%	8×10^-11^
*SLC6A5/TBX20*	97%	40%	52%	58%	2×10^-12^
**hrHPV detection**					
GP5+/6+ and Cobas	42%	80%	80%	72%	5×10^-3^

˘ linear-by-linear association test.

Analysis of hrHPV in the same scrapings revealed that hrHPV detection was more frequent when the underlying lesion was more severe (P = 0.005, see Table 3, Supplementary Table S5). Also, hrHPV was detected at similar rates in both ADC and SCC (75% vs. 68% respectively, P > 0.7, data not shown).

The hrHPV test classified samples differently than *SOX1*/*SOX14* hypermethylation (P < 10^-4^). In this population of women referred with abnormal cytology, methylation analysis was less sensitive to detect CIN2+ (P < 0.001), however, equally sensitive for CIN3+ (P > 0.2), and produced less false-positives compared to hrHPV analysis (P < 10^-5^).

## DISCUSSION

In our study, we used a genome-wide DNA methylation screening strategy detecting ADC as well as SCC with its precursor lesion in cervical scrapings. In addition, many differentially methylated regions were observed when normal cervices were compared with both ADC and SCC. The observation that some regions were differentially methylated exclusively in ADC or SCC could reflect exposure to different environmental factors [38]. Specifically, this observation may be explained by the difference in risk factors – smoking and high parity are risk factors for SCC [39] and obesity is a risk factor for ADC [40].

GO analysis pointed out that most of the pathways affected by hypermethylation in SCC were also affected in ADC, indicating similar pathways are deregulated by hypermethylation during carcinogenesis independent of histological cancer subtype. Pathways identified were all known to be involved in carcinogenesis [41–43]. Of the 53 differentially methylated candidates that were found in both ADC and SCC, 20 genes (Supplementary Table S3) were described previously in literature as being more frequently methylated in cancer, and 6 genes in (squamous-cell) cervical cancer (*SOX1*, *SOX14*, *ONECUT1* and *WT1*) [44] or high-grade CIN (*GFRA1*, *SOX1* and *TBX20*) [34, 45].

Compared to the gene panels recently reported by our group [34] the combination of *SOX1*/*SOX14* methylation showed less positive test results, both in scrapings from women without cervical disease and in women with high-grade CIN, but not in women with cervical cancer. Further research (e.g. by decision tree algorithms or latent structure analysis) is necessary to evaluate whether these markers are additive to each other. Furthermore, we cannot exclude the possibility that other interesting candidate genes are present in the highest ranking genes beyond the top 15 regions (Supplementary Table S3). Alternatively unmethylated CIN2+ samples may be analyzed by genome-wide methods to develop complementary assays that increase the clinical sensitivity. In addition, it can be hypothesized that the positive CIN2/3 samples are more similar to the cervical cancer lesions compared to the negative CIN samples; possibly reflecting the percentage of women which might develop cancer when left untreated.

In this study, we used MethylCap-seq to draw detailed methylome maps. Enrichment for methylated DNA by either MBD proteins (MethylCap) or antibodies (MeDIP) allows comparable distinction between methylated and unmethylated regions as bisulfite-based methods, but is less accurate to quantify the DNA methylation levels in partially methylated genomic regions. However, MethylCap is able to detect roughly twice as many DMRs compared to MeDIP at comparable sequencing depths [46]. Recent data showed that although MethylCap-seq was less sensitive compared to the array-based method of Infinium, more regions could be identified genome-wide [47]. Pyrosequencing did offer single-base resolution and could verify the MethylCap-seq results. Subsequently, primers for (Q)MSP were designed as these assays are more appropriate for high-throughput diagnostics. All five identified candidate markers discriminated between normal epithelium and cancer. A relatively good specificity was observed when using a threshold.

When we further validated the best four QMSPs on a series of scrapings of women referred with an abnormal smear, we observed that *SOX1* and *SOX14* provided a relatively good sensitivity, whereas *TBX20* and *SLC6A5* provided a relatively good specificity. Combining biomarker test results is a common choice to enhance the accuracy of clinical diagnosis [48]. Combining only *SOX1* and *SOX14* seems to be sufficient to provide the highest sensitivity and specificity. As to cervical cancer diagnostics, an important advantage of DNA methylation markers is that they can be tested on the same material as used for HPV analyses [49, 50]. When comparing the detection of disease using hrHPV testing with the gene combination *SOX1* and *SOX14*, we observed no difference in CIN3+ sensitivity, but a higher specificity in the methylation test. However, these findings need to be validated in population-based screening cohorts; particularly because this population is not representative of a referral population. Besides, the remaining 20% of CIN2+ samples are considered by us as hrHPV test-negative and not definitely hrHPV-negative [51]. Possibly these samples were infected with a HPV type that is not detected by the assays or infected with multiple types (e.g. high-risk and low-risk) leading to a reduced sensitivity [52].

So far only a limited number of methylated genes have been examined in ADC, especially using cervical scrapings in a large series. These studies revealed markers with a different clinical utility, i.e. with a lower sensitivity for both ADC and SCC or either one [24, 26, 27, 53–58]. Two genes of the Wnt pathway, *DKK3* and *SFRP2*, showed more methylation in ADC tissue compared to SCC tissue (82% vs. 18% and 84% vs. 39%) and combined analysis in cervical scrapes (n=8) detected all AdCIS and ADC [27]. Recently, *PAX1*, *PTPRR*, *SOX1* and *ZNF582*, previously reported to be frequently methylated in scrapings of SCC patients, were also analyzed in scrapings of ADC patients and showed a sensitivity of the single genes of 82% - 93% with a specificity of 81% - 95% in a Taiwanese population [26]. However, data on screening large cohorts with these markers is currently unavailable.

All of the 4 genes that we identified have previously been reported to be methylated in cancer. *SOX1* and *SOX14* belong to the SOX family of transcription factors having similar DNA binding specificities yet with divergent functions [59]. *SOX1* encodes a transcription factor implicated in the regulation of embryonic development and in the determination of cell fate. DNA methylation of *SOX1* in cervical cancer has been reported by Lai *et al*. [26, 44] albeit in a different region. Furthermore, *SOX1* was identified as a tumor suppressor gene, because it interfered with Wnt/β-catenin signaling in cervical cancer cells [60] and hepatocellular carcinoma [61]. Hypermethylated *SOX1* was also found in ovarian cancer cells that are chronically exposed to cisplatin [62]. *SOX1* methylation, at least in part, is responsible for cisplatin resistance in human non-small cell lung cancer (NSCLC) [25, 63]. *SOX14*, in contrast to our data, has been reported to be a potential marker to differentiate between ADC and SCC, with more methylation or mutation in SCC as determined by NotI-microarrays [55].

T-box (TBX) transcription factors belong to an ancient gene family with critical roles in embryogenesis, in early cell fate decisions and in control of differentiation and organogenesis [64]. *TBX20* methylation has previously been related to specific bladder cancers [65], late stage hepatocellular carcinoma development [66], recurrence of lung adenocarcinoma [67] and cervical cancer [45]. *TBX20* expression has been related to colorectal cancer [68].

So far, methylation of *SLC6A5* (also known as the glycine transporter gene *GLYT2*) was associated with glioma [69], prostate cancer [70, 71], oral and pharyngeal cancer [72]; and the expression was down-regulated during rat liver regeneration [73]. Additionally, the *SLC6A5* gene can form multigene complexes under the influence of TNF signaling, and is subsequently activated by the NF-κB transcription factor [74].

Overall, our approach resulted in four new cervical cancer methylation markers with high specificity and high sensitivity for both cervical ADC and SCC. These results indicate that especially *SOX1* and *SOX14* are meaningful for cervical cancer screening.

## MATERIALS AND METHODS

### General strategy

In order to identify and validate cervical cancer-specific methylation markers both for ADC and SCC, the following strategy was applied (schematically represented in Figure 1). Step 1, DNA from snap-frozen tissue of 12 cervical cancers (ADC=6, SCC=6) and 20 normal cervices was analyzed using MethylCap-seq. Subsequently, the differentially methylated regions (DMRs) were identified between normal cervices and both cancer subtypes. Step 2, among the methylation candidates, the top 15 was selected for verification by methylation-specific PCR (MSP) or pyrosequencing on the same frozen tissues from step 1. Step 3, using the selected candidates from step 2, MSP was performed on DNA of FFPE tissues from an independent series of 17 normal and 13 cancer samples (ADC=6, SCC=7). Step 4, the candidate regions that showed more methylation in cancer tissues were selected for further clinical validation by quantitative MSP (QMSP) on cervical scrapings from a large series of cervical cancer patients (n=125: comprising 57 ADC and 68 SCC) and 89 controls of comparable age. Step 5, the markers that best distinguished scrapings from normal cervices and cervical cancers were selected for a second diagnostic evaluation, provided the markers could sufficiently detect *both* ADC and SCC. QMSP was performed on scrapings of women referred with an abnormal smear and with known histological diagnosis (n=229: no CIN=27, CIN1=38, CIN2=45, CIN3=61, AdCIS=14 and (mi)Ca=44: ADC=12, SCC=29, adenosquamous=3).

### Patients

Patients with cervical cancer referred to the outpatient clinic of the University Medical Centre Groningen (UMCG) are asked to participate in our on-going ‘Methylation study’ that has been approved by the Institutional Review Board of University Medical Centre Groningen, the Netherlands. All patients from whom material was obtained gave written informed consent. Snap-frozen tissue, FFPE tissue and scrapings for this study are prospectively collected and stored in our tissue bank.

Within our ‘Methylation study’, normal tissue samples and normal scrapings are also collected from patients planned to undergo a hysterectomy for non-malignant reasons. All cervical tissue that was used for the normal control group was judged as histopathological normal. Additionally, all women without cervical disease never had an abnormal cervical smear prior to inclusion. Patients referred with cervical cancer are staged according to the FIGO criteria with pelvic examination and biopsies under general anesthesia. All cervical scrapings are collected prior to treatment. All cervical tissue samples were scored by an experienced gynecologic pathologist and the histological classification was used as the reference standard. All clinicopathological data were retrieved from patient files and stored in our large anonymous password-protected institutional Gynecologic Oncology database.

For MethylCap-seq and pyrosequencing, frozen tissue specimens were collected from 20 patients with a normal cervix (median age: 43 years, IQR 33-45) and 12 cancer patients (median age: 44 years, IQR 27-69) composed of 6 SCC and 6 ADC. For MSP analysis, FFPE tissue was collected from 17 patients with a normal cervix (median age: 43 years, IQR 40-44) and 13 cervical cancer patients (median age: 49 years, IQR 42-54) including 6 ADC and 7 SCC. For QMSP, scrapings were collected from 89 patients with normal cervices (median age: 47 years, IQR 43-53), and from 125 cervical cancer patients (median age: 49 years, IQR 39-63, P=0.32) comprising 68 SCC and 57 ADC. See Supplementary Table S6 for the histological classification, age and FIGO stage of all cervical cancer patients in this study.

Likewise, scrapings were selected from women in whom an abnormal cervical smear was found (n=229). These scrapings are prospectively collected from women referred to our outpatient clinic for colposcopy after being tested with an abnormal smear in the population-based screening program. Here we randomly selected scrapings – i.e. within each diagnosis group – from 27 women diagnosed without CIN (median age: 36, IQR 30-48), 38 with CIN1 (median age: 40, IQR 31 to 45), 45 CIN2 (median age: 35, IQR 31-40), 61 CIN3 (median age: 35, IQR 32-40), 14 adenocarcinoma in situ (AdCIS) (median age: 36, IQR 32-44) and 44 with (micro-invasive) carcinoma (median age: 40, IQR 36-50). Of the AdCIS scrapings 3 were collected at the Meander Medical Centre (Amersfoort, the Netherlands) and 6 at the VU University Medical Centre (Amsterdam, the Netherlands) [75, 76]. The (mi)Ca cases comprised 12 ADC (median age: 40, IQR 36 to 49), 29 SCC (median age: 40, IQR 36 to 50) and 3 adenosquamous carcinomas (see Supplementary Table S5).

### Sample collection and DNA isolation

Preparation of tissue slides, enrichment for epithelial (tumor) cells by macrodissection, collection and processing of cervical scrapings, the DNA isolation and the assessment of the DNA's structural integrity were as described previously [22, 23, 77, 78]. DNA concentrations and 260/280 ratios were measured using a Nanodrop ND-1000 spectrophotometer (Thermo Scientific, Waltham, MA, USA). A 260/280 ratio around 1.8 and the capability to produce amplicons of at least 300 base pairs (bp) was required for all DNA samples. For the MethylCap-seq samples, DNA concentrations were measured using the Quant-iT™ PicoGreen^®^ dsDNA Assay kit (Invitrogen, Carlsbad, CA, USA).

### MethylCap-seq

To assess genome-wide methylation patterns, MethylCap-seq was performed at the University of Ghent using the MethylCap kit according to manufacturer's instructions (Diagenode, Liège, Belgium) as previously described [33, 34]. Briefly, DNA samples (500 ng) were sheared to a size range of 300 - 1000 bp using a Bioruptor™ UCD-200 (Diagenode, Liège, Belgium) and fragments of approximately 300 bp were isolated and subsequently captured. Captured DNA was paired-end-sequenced on the Illumina Genome Analyzer II platform according to protocol (Illumina, San Diego, CA, USA). Leukocyte DNA of 4 healthy women was also included in 2 sets of 2 samples. Results were mapped using Bowtie software [79], visualized using BioBix' H2G2 browser (http://h2g2.ugent.be/) and processed using the human reference genome (NCBI build 37). The paired-end fragments were unique and located within 400 bp of each other [80].

### MethylCap-seq analysis

Read data of the promoters and exons were retrieved and dichotomized into methylation positive (if ≥ 3 reads) or methylation negative (if 0 or 1 read). Subsequently, Fisher's exact test was performed to identify DMRs between ADC and normal and between SCC and normal. To downsize the number of DMRs and to pinpoint candidate methylation markers in cervical cancer the following additional criteria were applied (depicted in Figure 2): 1) at least 75% (15/20) of the normal cervix group was methylation negative; 2) at least 50% (3/6) of ADC as well as at least 50% (3/6) of SCC was methylation positive; 3) no methylation in leukocytes, i.e. no more than 1 read for both sample pools or more than 2 reads for any individual leukocyte pool; 4) the region is at least 30 bp long.

### Gene ontology analysis

The Ensembl gene identifiers that were coupled to the DMRs were used for functional classification and GO analyses using DAVID v6.7 [81] including the annotated hypermethylated DMRs in ADC or SCC.

### Bisulfite treatment

Sodium bisulfite modificationof denatured genomic DNA was performed as previously reported [82]. One microgram of genomic DNA per sample was converted using the EZ DNA methylation kit (Zymo Research Corp, Irvine, US-CA). Leukocyte DNA from healthy women and WGA (illustra ready-to-go GenomiPhi HY kit, GE healthcare, Little Chalfont, UK), were used as negative controls for methylation, whereas *in vitro* methylated leukocyte DNA, produced using M. SssI methyltransferase (New England Biolabs, Ipswitch, US-MA), served as a positive control.

### Pyrosequencing

Bisulfite-modified DNA (BS-DNA) was amplified using PyroMark PCR kit reagents and conditions (Qiagen, Hilden, Germany), yet we used a universal biotinylated primer as previously described [33, 83]. Sample preparation and pyrosequencing was performed on a PyroMark Q24 platform using PyroGold Q24 reagents (Qiagen, Hilden, Germany). Experiments were designed with PyroMark Assay Design 2.0; primers were checked for specificity with BiSearch [84] and sequences are available upon request. Non-template control (water), positive and negative controls were used in each reaction.

### Methylation-specific PCR

Each reaction was performed in 30 μl total reaction volume, containing 600 nM of each primer, 1.5 μl BS-DNA (approximately 15 ng), and 0.5 U AmpliTaq Gold DNA polymerase (Applied Biosystems, Carlsbad, CA, USA). The thermal profile of the MSP was 10’ hot-start at 95°C, 40 cycles of 95°C for 60”, 60°C for 60”, 72°C 60”, and finally an elongation step of 7’ at 72°C. PCR products were separated on a 2.5% agarose gel, pre-stained with 0.5 μg/ml ethidium bromide and visualized by UV transillumination. Non-template control (water) and positive/negative controls were used in each reaction. MSP primers were designed with Methyl Primer Express version 1.0 (Applied Biosystems, Carlsbad, CA, USA), were checked for specificity with BiSearch [84] and are available upon request. Separate reactions were performed to detect either unmethylated or methylated template.

### Quantitative methylation-specific PCR

QMSP was performed as we described previously [22] with a double-quenched hybridization probe (Integrated DNA Technologies, Leuven, Belgium). Probe sequences are available upon request. The *ACTB* gene was used as a methylation independent reference reaction. QMSP was performed in 10 μl containing 300 nM of each primer, 200 nM probe, QuantiTect Probe PCR Master Mix (Qiagen, Hilden, Germany) and 2.5 μl BS-DNA (approximately 25 ng). Each sample was analyzed in triplicate by ABI PRISM^®^ 7900HT Sequence Detection System (Applied Biosystems, Carlsbad, CA, USA). Serial dilutions of *in vitro* methylated leukocyte DNA enabled absolute quantification of (methylated) template. A DNA sample was considered methylated if at least 2 out of the 3 wells were methylation positive with a C_q_ below 50 with at least 225 pg *ACTB* input. The relative level of methylation of the region of interest was expressed as: (average quantity of methylated DNA / average quantity of *ACTB*) x 10000 [85].

### High-risk HPV testing

The presence of clinically relevant levels of hrHPV DNA was assessed using GP5+/6+ PCR and subsequently by Cobas HPV PCR as described previously [34]. The 6 AdCIS samples that were collected at the VU University Medical Centre (Amsterdam, the Netherlands) were previously tested hrHPV-positive with a GP5+/6+ enzyme immunoassay [86].

### Statistical analysis

Statistical analysis was performed using IBM SPSS Statistics 22 (IBM Corporation, New York, US-NY). Chi-square test and Fisher's exact test for small numbers were used to analyze the different methylation frequency between normal and cancer. The average methylation level of each frozen tissue sample and MethylCap-seq reads were correlated using Spearman's rank test. The Mann-Whitney U test was used to determine differences in median methylation levels between 2 groups. The sensitivity, specificity, ROC curves and area under the ROC curve (AUC) were calculated for the first diagnostic evaluation (normal vs. cervical cancer) [87]. The optimal threshold was calculated based on the largest Youden's index J [88, 89]. The Jonckheere-Terpstra test was used to assess whether the methylation levels changed with the severity of the underlying lesion. The chi-square linear-by-linear test was applied to analyze the dichotomous results across histological types. The McNemar-Bowker test was employed to assess differences in test classification, and the McNemar χ^2^ test was subsequently used to attribute differences to either sensitivity or specificity or both. A gene combination labeled a sample positive if at least one of those QMSPs produced a positive test. A P-value below 0.05 was considered to be significant.

## SUPPLEMENTARY MATERIALS TABLES














