# Supplementary material for: Genome-wide methylome analysis using MethylCap-seq uncovers 4 hypermethylated markers with high sensitivity for both adeno- and squamous-cell cervical carcinoma
Source: Oncotarget. 2016 Oct 12;7(49):80735–50. doi: 10.18632/oncotarget.12598 (PMC5348351; doi:10.18632/oncotarget.12598)
Supplement: Supplementary file 1 [file oncotarget-07-80735-s001.pdf]

Genome-wide methylome analysis using MethylCap-seq uncovers 4 hypermethylated markers with high sensitivity for both adeno- and squamous-cell cervical carcinoma

SUPPLEMENTARY TABLES

Supplementary Table S1: Gene ontology categories of the 446 candidate DMRs in the ADC group (328 GO terms)

See Supplementary File 1

Supplementary Table S2: Gene ontology categories of the 93 candidate DMRs in the SCC group (49 GO terms)

See Supplementary File 2

Supplementary Table S3: The top 15 markers of the 53 identified candidate DMRs. The ranking first ensured high amount of positive ADC, then positive SCC and last negative normals

See Supplementary File 3

Supplementary Table S4: Overview of genes that endured the different stages of the appraisal process.

See Supplementary File 4

Supplementary Table S5: Diagnostic performance of individual genes, gene combinations and hrHPV analysis in cervical scrapings of women referred with an abnormal smear (presented as test positive fraction of total and ranked on sensitivity CIN3+)

See Supplementary File 5

Supplementary Table S6: Histological classification, age and stage of all cervical cancer patients in this study

See Supplementary File 6
